# TCOF1 pathogenic variants identified by Whole-exome sequencing in Chinese Treacher Collins syndrome families and hearing rehabilitation effect

**DOI:** 10.1186/s13023-019-1136-z

**Published:** 2019-07-15

**Authors:** Xinmiao Fan, Yibei Wang, Yue Fan, Huiqian Du, Nana Luo, Shuyang Zhang, Xiaowei Chen

**Affiliations:** 1 Department of Otolaryngology, Peking Union Medical College Hospital, Peking Union Medical College and Chinese Academy of Medical Sciences, Beijing, China; 2 Allwegene Technology Inc, Tianjin, China; 3 Allwegene Technology Inc, Beijing, China; 4 Department of Cardiology, Peking Union Medical College Hospital, Peking Union Medical College and Chinese Academy of Medical Sciences, Beijing, China

**Keywords:** Treacher Collins syndrome (TCS), Whole-exome sequencing, *TCOF1*, Bone conduction hearing rehabilitation

## Abstract

**Background:**

Treacher Collins syndrome (TCS, OMIM 154500) is an autosomal disorder of craniofacial development with an incidence rate of 1/50,000 live births. Although *TCOF1*, *POLR1D*, and *POLR1C*, have been identified as the pathogenic genes for about 90% TCS patients, the pathogenic variants of about 8–11% cases remain unknown. The object of this study is to describe the molecular basis of 14 clinically diagnosed TCS patients from four families using Whole-exome sequencing (WES) followed by Sanger sequencing confirmation, and to analyze the effect of bone conduction hearing rehabilitation in TCS patients with bilateral conductive hearing loss.

**Results:**

Four previously unreported heterozygous pathogenic variants (c.3047-2A > G, c.2478 + 5G > A, c.489delC, c.648delC) were identified in the *TCOF1* gene, one in each of the four families. Sanger sequencing in family members confirmed co-segregation of the identified TCOF1 variants with the phenotype. The mean pure-tone threshold improvements measured 3 months after hearing intervention were 28.8 dB for soft-band BAHA, 36.6 ± 2.0 dB for Ponto implantation, and 27.5 dB SPL for Bonebridge implantation. The mean speech discrimination improvements measured 3 months after hearing intervention in a sound field with a presentation level of 65 dB SPL were 44%, 51.25 ± 5.06, and 58%, respectively. All six patients undergoing hearing rehabilitation in this study got a satisfied hearing improvement.

**Conclusions:**

WES combined with Sanger sequencing enables the molecular diagnosis of TCS and may detect other unknown causative genes. Bone conduction hearing rehabilitation may be an optimal option for TCS patients with bilateral conductive hearing loss.

## Background

Treacher Collins syndrome (TCS, OMIM 154500) is an autosomal disorder of craniofacial development that has an incidence rate of 1/50,000 live births [1, 2]. TCS is characterized by typical bilateral craniofacial malformations, such as hypoplasia of the mandible and zygomatic complex, downward-slanting palpebral fissures, coloboma of the lower eyelids, antimongoloid slant of the eyes, micrognathia, cleft palate and microtia, and most cases are associated with conductive hearing loss [3], which affect patients both in cosmetic and functional aspects. Diagnosis and subsequent genetic counseling may be very difficult because some individuals are only mildly affected, and there are clinical overlaps between TCS, Goldenhar syndrome, Miller syndrome and Nagar syndrome, all of which are thought to be caused by impaired development of the first and second branchial arches between the 5th and 8th weeks of embryonic development.

Historically, a diagnosis of TCS has been based on the clinical identification of a minimal clinical phenotype: downward slanting palpebral fissures and hypoplasia of the zygomatic arch. However, this can overlook some mildly affected patients. The use of molecular diagnosis could enable the range of TCS phenotypes to be determined with less bias [4]. TCS is genetically heterogeneous, having been associated with pathogenic changes in three causative genes: *TCOF1* (OMIM 606847), *POLR1D* (OMIM 613715), and *POLR1C* (OMIM 610060). More than 200 distinct mutations have been reported in *TCOF1*, accounting for about 70–93% of TCS individuals, which are inherited via an autosomal dominant pattern, while *POLR1D* and *POLR1C* mutations have been found in about 11–23% of the remaining patients, which are inherited via autosomal dominant and autosomal recessive patterns, respectively [3, 5–7].

To date, molecular diagnosis of TCS has focused on Sanger sequencing of these three known pathogenic genes, a method that is now the recommended first-tier test for TCS. The causative pathogenic variants of ~ 8–11% of TCS cases remain unknown, suggesting that there may be other TCS-related genes [8]. No phenotype-genotype correlation has been found in TCS patients [3]. Although nonpenetrance is rare, there is high inter-and intra-familial phenotypic variation, ranging from mildly affected cases to perinatal death due to severe craniofacial malformations that cause airway obstruction [3, 9]. With the development of next generation sequencing (NGS) technology, the cost of Whole-exome sequencing (WES) has become progressively lower in the past few years. WES could help to screen new causative genes compared to Sanger sequencing of *TCOF1*, *POLR1D*, and *POLR1C*. In this study, we used WES combined with Sanger confirmation to screen for causative genes in TCS families in China.

The occurrence rate of TCS in China is low, which has hindered genetic counseling for Chinese TCS patients. Although there have been several genetic studies of TCS in Chinses populations [8, 10, 11], most of the enrolled TCS cases are sporadic. Here, we describe four Chinese families containing 14 TCS patients. We performed WES in the four probands of these unrelated families and identified one previously undescribed pathogenic *TCOF1* variant in each family, followed by Sanger sequencing to perform familiar segregation analysis. Our findings provide relevant information to diagnose TCS patients and counsel their families.

TCS is not a progressive disease. The primary concern in a newborn TCS patient is respiratory failure arising from craniofacial malformation-associated airway narrowing. Early interventions may be required to clear and maintain the airway, enable feeding, protect the eyes, improve the auditory ability, and allow for the development of speech. Later operations may include aesthetic and functional reconstructions of the mouth, face, and external ear [12]. With respect to the ear, 50% of TCS patients suffer from anomalies of the middle ear ossicular chain and a size reduction of the middle ear cavity, which can lead to bilateral conductive hearing loss. Bone conduction hearing aids or middle ear surgery are usually used to improve hearing in these patients [13, 14]. In the families studied herein, various hearing interventions were conducted for six TCS patients suffering from bilateral conductive hearing loss. We evaluated and compared their effects.

## Results

### Patients

The present study included nine female and four male patients from four families, each of which included at least two patients and were of Han nationality. The major clinical features of all patients were evaluated (Table 1). Downward slanting palpebral fissures and mandibular hypoplasia were observed in all patients. All patients had conductive hearing loss of different degrees. For the six patients that underwent hearing intervention during the study period, the average air-conducted hearing thresholds ranged from 56.25 dB HL to 60 dB HL and the bone-conducted hearing thresholds were ≥ 30 dB HL at frequencies of 0.5–4 kHz. HRCT scans demonstrated hypoplasia of the facial bones in all patients, including the zygomatic arch, mandible, and external ear canals. Temporal bone CT revealed malformation of the ossicles with fusion between rudiments of the malleus and incus.

**Table 1:** Phenotype of TCS probands

Patients	Sex	Family history	Downward slanting palpebral fissures	Lower eyelid coloboma	Zygomatic hypoplasia	Mandibular hypoplasia	Microtia	Atresia of external ear canal	Conductive hearing loss	Delayed speech development
2313	F	–	+	+	+	+	–	–	+	–
3538	M	–	+	+	+	+	–	–	+	+
2848	M	–	+	+	+	+	+	+	+	–
2721	F	+	+	–	+	+	+	+	+	+
3314	F	+	+	+	+	+	–	–	+	+
3316	F	+	+	+	+	+	–	–	+	–
3286	M	+	+	+	+	+	–	–	+	+
3287	M	+	+	+	+	+	–	–	+	+
3288	M	+	+	+	+	+	–	–	+	+
3289	F	+	+	–	+	+	–	–	+	–
3290	F	+	+	+	+	+	–	–	+	–
3291	F	+	+	–	+	+	–	–	+	–
3292	F	+	+	+	+	+	+	–	+	–
3293	M	+	+	–	–	+	–	–	+	–

### Pathogenic variants

**Fig. 1:**
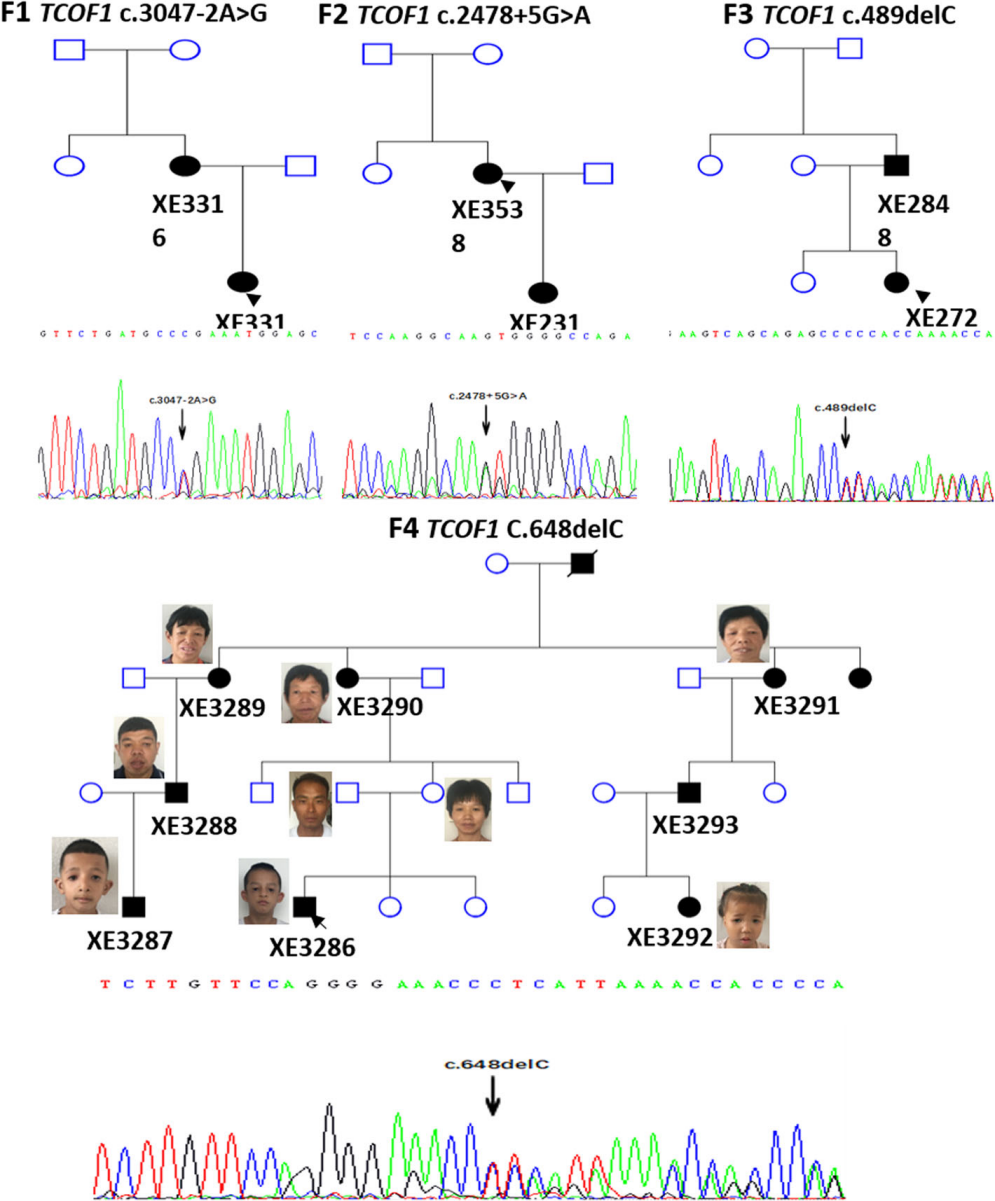
(F1) Sequence of the patients of Family 1 showed a heterozygous mutation c.3047-2A > G. (F2) Sequence of the patient of Family 2 showed a heterozygous mutation c.2478 + 5G > A. (F3) Sequence of the patient of Family 3 showed a reported mutation c.489delC. (F4) Sequence of the patients of Family 4 showed a heterozygous mutation c.648delC

**Fig. 2:**
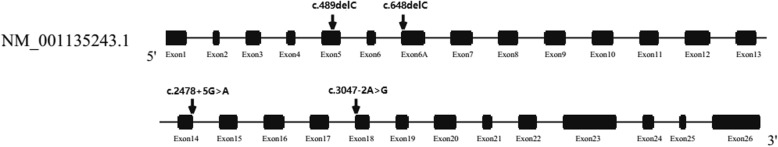
Spectrum of causative mutations in the *TCOF1* gene (NM_001135243.1) in our patients. Coding exons are proportionately represented by black boxes. Introns are not scaled. Mutations are marked with arrows

Four different and previously undescribed pathogenic variants of *TCOF1* were identified in the four families (Fig. 1): c.3047-2A > G, c.2478 + 5G > A, c.489delC, and c.648delC, corresponding to two deletion mutations and two splicing mutations. The mutational spectrum of the four families are shown in Fig. 2. Sanger sequencing confirmed that all affected family members carried the relevant pathogenic mutation, whereas their unaffected relatives did not. The pathogenic variants found in this study are presented in Table 2.

**Table 2:** TCOF1 pathogenic variants in Chinese individuals with TCS

Family	Subjects	Nucleotide	Protein	Exons	Predict effect	Reference
1	3314, 3316	c.3047-2A > G	p.(?)	Intron 17	Splice	This report, 1^a^
2	3538, 2313	c.2478 + 5G > A	p.(?)	Intron 14	Splice	This report, 1^a^
3	2721,2848	c.489delC	p.S164Qfs*55	5	Frameshift	This report
4	3286, 3287, 3288,3289, 3290, 3291, 3292, 3293	c.648delC	p.S217Qfs*2	6A	Frameshift	This report

^a^The location of the variants occurred were same as the reference, but the nucleotide changes were different

### Hearing improvement

**Fig. 3:**
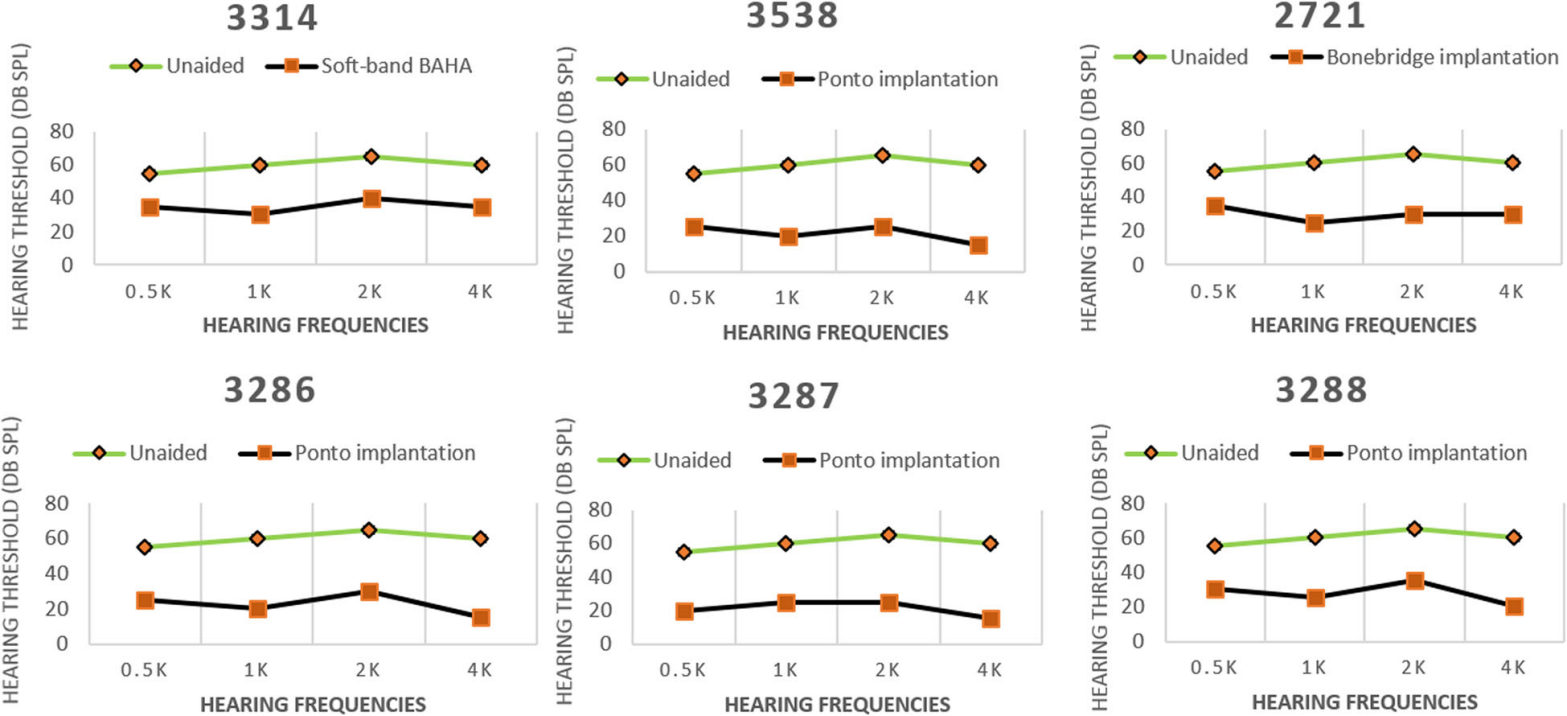
The frequency specialized hearing thresholds unaided and with bone conduction aid of the six TCS patients

**Fig. 4:**
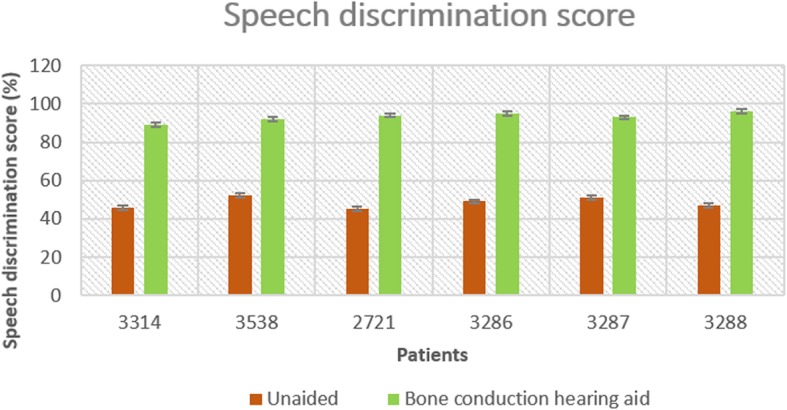
The speech discrimination scores unaided and with bone conduction aid of the six TCS patients. 3314: Soft-band BAHA; 3538, 3286, 3287, 3288: Ponto implantation; 2721: Bonebridge implantation

The mean pure-tone threshold improvements measured 3 months after hearing intervention were 28.8 dB for soft-band BAHA, 36.6 ± 2.0 dB for Ponto implantation, and 27.5 dB for Bonebridge implantation. The frequency specialized hearing thresholds unaided and with bone conduction hearing aid of the six patients were shown in Fig. 3. The mean speech discrimination improvements measured 3 months after hearing intervention in a sound field with a presentation level of 65 dB SPL were 44%, 51.25 ± 5.06, and 58%, respectively. The speech discrimination scores of each patient unaided and with bone conduction hearing aid were shown in Fig. 4. All six patients undergoing hearing rehabilitation in this study got a satisfied hearing improvement.

## Discussion

TCS is caused by abnormal formation of the first and second branchial arches during the 5th to 8th weeks of fetal development, which leads to profound facial dysmorphism. Phenotypic expressivity varies between families and within families, and the disease is known to be both genetically and phenotypically heterogeneous. No phenotype-genotype correlation has been established to date. About 60% of patients arises as the result of de novo mutations without family history of this disease. Although there have been several genetic studies of TCS in Chinese populations [2, 8, 10], most of the enrolled TCS cases are sporadic. Family studies allow segregation analysis to support the identified genetic variants as pathogenic. We report the molecular characterization and bone conduction hearing rehabilitation effect on four Chinese TCS families, including a four- generation family with ten affected members.

Molecular diagnosis is of great significance for TCS families which can provide information for their genetic counseling. Han et al. (2018) reported the clinical findings and molecular diagnostics of a Chinese family with TCS. They concluded that the progeny of the proband and her mother have a 50% risk of suffering from TCS and therefore genetic counseling is necessary [15]. However, their study enrolled only one TCS family focused on molecular diagnosis using Sanger sequencing of TCOF1 and did not give any intervention to the patients. *TCOF1, POLR1C*, and *POLR1D* have been identified as causative genes for TCS, but mutations in these genes have not been found in ~ 8–11% of TCS cases. This, along with the time-consuming nature of Sanger sequencing, has limited the direct analysis of these genes for the systematic molecular diagnosis of TCS [4, 16]. Considering that the cost of WES has become progressively lower in the past few years, we used WES to detect the new causative gene in four TCS families. Although the identified mutations were in the known *TCOF1* gene, it’s still of significance for the molecular diagnosis of TCS families.

Over 50% of TCS patients have bilateral conductive hearing loss due to abnormal development of the external/middle ear [12, 17]. These patients often require a multidisciplinary treatment approach that includes hearing intervention. In this study, hearing rehabilitations were performed for six patients with bone conductive hearing loss. We evaluated and compared the outcomes. This work, which describes clinical and molecular aspects of 14 Chinese TCS patients, and evaluates the effect of bone conduction hearing aids in TCS patients, could help facilitate the molecular diagnosis and treatment of TCS.

### Mutations detected in this study

Most cases of TCS represent an autosomal dominant disorder of craniofacial development. Positional cloning enabled researchers to identify *TCOF1* as the major causative gene, and a series of mutations within its coding sequence have been identified [18]. The most common causative mutations in TCS are small deletions (60%) and duplications (25%), all of which lead to frameshift variations [19]. Splicing, missense, and nonsense mutations are also seen, the vast majority of which are predicted to introduce a termination codon into the mRNA [17, 18, 20, 21]. The mutations associated with TCS are consistently predicted to shorten treacle, which is the gene product of *TCOF1*, and thus might cause dominant-negative effects. Alternatively, it has been suggested that mutation of one *TCOF1* allele may cause TCS by haploinsufficiency [22, 23]. In addition to these mutations in *TCOF1*, mutations in genes encoding two subunits of *POLR1C* and *POLR1D* have also been associated with TCS [6].

The 14 patients studied herein were all found to have pathogenic variants in *TCOF1*. The high detection rate of pathogenic variants achieved in this study suggests that WES combined with Sanger sequencing may be a useful method for detecting pathogenic variants in TCS families. Four different pathogenic *TCOF1* variants were identified and all haven’t been previously reported, which broadens the mutational spectrum in the Asian population. Among the studied patients, 71.4% (10/14) had one nucleotide deletion that were predicted to yield haploinsufficiency of the treacle protein. This finding is consistent with most of the previous studies. In the present work, each family harbored a different mutation. The most frequent mutation was a deletion (C. 648delC) in exon 6A, which was found in eight patients of the same family. Although the literature suggests that exons 10, 15, 16, 23, and 24 are mutational hotspots in *TCOF1*, the mutations identified in the present work were located in exon 6A, exon 5, intron 14, and intron 17 [7, 24, 25].

The proband of one family (F4) was initially suspected to have autosomal recessive (AR) inheritance at the time of clinical diagnosis, as both of his parents showed normal physical features. Our work ruled out this potential AR inheritance, however, as we found that the proband’s seemingly normal mother carried a 1-bp deletion C in exon 6A of *TCOF1*, as did her affected mother and other affected individuals of this family. The clinical diagnosis was missed in the proband’s mother because she showed mild downslanting palpebral fissures that were rendered nearly inconspicuous by the droop of her aging eyelids. This serves to emphasize that the severity of the TCS phenotype is highly variable, and some subjects are so mildly affected that it is almost impossible to make a clinical diagnosis without molecular analysis.

### TCOF1 mutation spectrum in TCS patients

The majority of TCS patients are heterozygous for mutations in *TCOF1*, which is located at 5q32–q33.1.11–18 and has an open reading frame that encodes 4465 base pairs and 28 exons. The gene product, treacle, contains at least 1411 amino acids and acts as a nucleolar phosphoprotein that travels between the nucleolus and the cytoplasm. Treacle is a low-complexity protein with a 14-residue N-terminus followed by 11 repeated units with potential phosphorylation sites and a C-terminus with multiple putative nuclear and nucleolar localization signals. It has been suggested that correct expression of treacle is essential for the survival of cephalic neural crest cells. The pathogenic *TCOF1* mutations can reduce the number of neural crest cells (NCCs), which are needed for craniofacial embryological development, by impacting the involvement of treacle in ribosomal DNA gene transcription. Nonsense mutations of *TCOF1* can lead to an immediate termination of translation, producing a shortened protein. The location of the mutation affects the length of the produced protein, and all of the shortened proteins are likely to be degraded via nonsense-mediated decay. In addition, the C-terminus of treacle contains multiple putative nuclear localization signals, which can be disrupted by two constructs that split the C-terminal region [20, 26, 27]. In the present study, we identified four mutations, including two deletions and two splicing mutations, all of which could lead to the production of a shortened treacle protein.

No clear phenotype-genotype correlation is seen in TCS, but the severity is related to the type of mutation. Although penetrance is high, there is intra- and interfamilial variation. 11–23% of patients have mutations in *POLR1C* or *POLR1D*, which encode proteins that are important in the ribosomal transcription of RNA and affect ribosomal biogenesis [6]. However, in this study we did not identify any mutation in *POLR1C* or *POLR1D*.

### Advantages of WES in identifying pathogenic variants

Most suspected cases of TCS can be molecularly confirmed by Sanger sequencing of three causative genes: *TCOF1*, *POLR1D*, and *POLR1C*. The major causative gene, *TCOF1*, has a total of 27 coding exons and adjacent splice junctions, rendering such analysis time-consuming and costly. We thus set out to use WES to quickly screen the causative exons, followed by Sanger sequencing of specific exons that appeared to confirm mutations.

The pathogenic variants in about 8–11% of TCS cases remain undetected. There are four main possible explanations for this. First, some of these cases may have been clinically misdiagnosed. However, we note that in most such cases, CT scans and clinical analysis have been used to confirm the diagnosis. Second, the causative mutations might be located in untranslated (and thus unexamined) regions of the three known TCS genes. Although such pathogenic variants are rare in the literature, these regions should be checked in future studies. Third, the causative mutations may be large deletions or insertions within the known TCS genes, which may not be detected by Sanger sequencing. This is especially true in dominant diseases, in which patients also possess the normal allele. Finally, there may be additional, yet-undiscovered genes responsible for TCS. These could potentially be identified by WES.

### Molecular diagnosis of syndromes with overlapping phenotypes

TCS, Goldenhar syndrome, Miller syndrome, and Nagar syndrome exhibit overlaps in their variable phenotypic expression, asymmetric involvement of facial structures, and familial occurrence of microtia or related anomalies (e.g., preauricular tags and pits). This complicates the diagnosis of such diseases according to the patient’s clinical manifestation. WES could help overcome this limitation. The common phenotypes and causative genes of these syndromes are shown in Table 3. The WES allowed us to exclude the involvement of these non-TCS causative genes and confirmed the molecular diagnosis of TCS in the 14 enrolled patients. It is helpful to conduct WES for clinical tentative TCS patients to identify the pathogenic variants and to differentiate from other syndromes sharing common clinical features.

**Table 3:** Phenotypes and related genetic factors of several similar syndromes

Syndromes	Phenotypes	Related genetic factors
Treacher Collins	Facial asymmetry; Eye antimongoloid slant; Lower eyelids colobomas; Microtia	*TCOF1, POLR1D, POLR1C*
Miller	Facial asymmetry; Lower eyelids colobomas; Micrognathia; Orofacial clefts; Postaxial limb defects	*DHODH*
Nager	Facial asymmetry; Anterior limb defects; Micrognathia; Midface retrusion; Cleft palate; Microtia	*SF3B4*
Goldenhar	Facial asymmetry; Microtia; Ear and facial tags; Epibulbar dermoids; Microphthalmia; Macrostomia	*GSC*

### Intervention for TCS patients

TCS is characterized by a complex presentation of mandibulofacial dysplasia that requires a multidisciplinary intervention from birth to adulthood. Although the presenting features are predictable, there is considerable individual variability, and the functional, aesthetic, and psychosocial needs of each patient will differ. Patients can be given a general scheme of operations and a broad description of the potential help that is available, but a more individually tailored approach is needed [12–14, 28]. Bilateral conductive hearing loss is seen in 50% of TCS patients, arising from a wide range of anomalies of the middle ear ossicular chain and a reduction in the size of the middle ear cavity. Deformities in the ossicular chain can be corrected surgically if the external meatus is patent; otherwise, bone conduction hearing aids are usually used [12]. Bone conduction hearing device implantation surgery requires that the cranial bone have a thickness of at least 4 mm, which is usually achieved at 6 years of age. Prior to this, patients are given banded bone conduction hearing aids. This is ideally initiated before 12 months of age, to enable proper central auditory neurological development. As CT may be indicated to assess the status of the middle ear and external meatus, the patient’s craniofacial and auditory ear, nose, and throat (ENT) teams should discuss their scanning protocols and intentions to ensure minimal exposure to radiation over time. The present study included six patients with bilateral conductive hearing loss who were given hearing interventions during the study period. All obtained optimal results.

It has been suggested that genetic or pharmacological blockade of the p53 gene could reduce neuroepithelial apoptosis during embryogenesis and restore the migrating population of NCCs, potentially preventing the TCS phenotype. However, this would also block the ability of p53 to act as a tumor suppressor, so it would be necessary to interrogate its downstream targets to find a safe point for intervention. This would have to occur in the first trimester, which would make it challenging to detect the need for and properly time the treatment [12, 29].

## Conclusions

We herein show that mutational analysis based on WES was useful for the definitive diagnosis of TCS Chinese families and may also provide more information for molecular diagnosis. We also report that bone conduction hearing rehabilitation was consistently helpful for TCS patients with bilateral conductive hearing loss.

## Methods

### Patients and families

This single-center prospective study, which involved four Chinese families with 14 clinically diagnosed TCS patients, was performed at Peking Union Medical College Hospital (PUMCH) in Beijing, China. Approval was obtained from the Institutional Review board of PUMCH and written informed consent was obtained from each studied family member. A comprehensive clinical history was taken and a complete physical examination was performed on all subjects to exclude Goldenhar, Nager, and Miller syndromes. The scoring system developed by Ozge Altug and Teber was used to clarify the phenotypic expression of TCS in these patients [4, 9]. All patients were identified as severely or mildly affected. Of the 14 patients, six underwent hearing rehabilitation between January 2017 and January 2018. All six patients received hearing measurements consisting of pure tone auditory (PTA) testing at 0.5, 1, 2, 4 kHz before and after hearing intervention. Clinical data, photographs of the patients, and temporal bone high-resolution computed tomography (HRCT) data were collected.

### WES and mutational analysis

Genomic DNA was extracted from peripheral blood samples using a TIANamp Blood DNA Kit (Tiangen, Beijing, China) according to the manufacturer’s protocol. WES was performed on the four TCS probands at Beijing Allwegene (Beijing, China). Exome enrichment was performed using a Sure Select Human All Exon v6 kit (65 Mb) (Agilent, Santa Clara, CA, USA), which yielded an average sequencing depth of 100-fold and a coverage of 97.7%. Enriched shotgun libraries were sequenced on a HiseqX platform (Illumina, San Diego, CA, USA).

Sequenced reads were collected, filtered for quality, and aligned to the human reference sequence (UCSC Genome Browser hg19, http://genome.ucsc.edu/) using the Burrows-Wheeler Aligner. Genotypes were called using SAMtools, Picard, and GATK. Sequence variants, including single-nucleotide variants (SNVs) and small insertions or deletions (InDels), were annotated using the ANNOVAR software (http://annovar.openbioinformatics.org) (TCOF1 reference: NM_001135243). For coding or splice-site mutations, the conservation at the variant site and the predicted effect on protein function were evaluated using the in silico tools, SIFT (http://sift.jcvi.org/), PolyPhen-2 (http://genetics.bwh.harvard.edu/pph2/), MutationTaster (http://www.mutationtaster.org/), and CADD (http://cadd.gs.washington.edu/).

A list of qualifying genotypes was generated using the following criteria: First, only protein-altering variants, such as missense variants, frameshift, InDels, and intron-exon boundary variants were included. Second, mutations were excluded as common variants if they were present at a frequency of 10% or more in at least one of the following databases: dbSNP (v.144); the 1000 Genomes Project; the HapMap CHB (Han Chinese in Beijing, China) population; the National Heart, Lung, and Blood Institute Exome Sequencing Project (ESP); and the Exome Aggregation Consortium (ExAC) Browser. Finally, missense variants were excluded if they were not predicted to be deleterious by the SIFT, PolyPhen-2, MutationTaster, or CADD analyses.

The mutations identified in the four families were prioritized for Sanger confirmation. The relevant sequences were PCR amplified from the probands and their family members, and the amplified fragments were purified using an Agencourt AMPure XP kit (Beckman Coulter, USA). Sanger sequencing was performed with an ABI3730xl DNA Sequencer (Applied Biosystems | Thermo Fisher Scientific, USA) and the results were analyzed using the Sequencing Analysis 5.2 software (Applied Biosystems | Thermo Fisher Scientific, USA). We referred to the HGVS nomenclature guidelines (http://www.hgvs.org/mutnomen) when naming the identified variants.

### Hearing interventions and audiometric data

Of the 14 TCS patients, six were given hearing interventions: one received a soft-band bone anchored hearing aid (BAHA), four received Ponto implantation, and one received bonebridge implantation. Pure-tone audiograms and speech discrimination tests were performed before and after the hearing interventions. Loudspeakers were placed 1 m in front of each subject and free sound field hearing thresholds were evaluated at 0.5, 1, 2 and 4 kHz frequencies. Speech discrimination scores (in quiet) were measured using the Mandarin Speech Test Materials (MSTM) [30], which comprised 10 lists of 50 Chinese characters or spondaic words. Speech stimuli were presented at 65 dB SPL. All test materials were presented without repetition. The average hearing gains at 0.5, 1, 2, and 4 kHz were calculated.

## Data Availability

All data generated during this study are included in this published article [and its supplementary information files.

## Abbreviations

AR: Autosomal recessive
TCS: Treacher Collins syndrome
WES: Whole-exome sequencing
